# Airborne Endotoxin Is Associated with Respiratory Illness in the First 2 Years of Life

**DOI:** 10.1289/ehp.8142

**Published:** 2005-11-03

**Authors:** Robert Dales, David Miller, Ken Ruest, Mireille Guay, Stan Judek

**Affiliations:** 1 Air Health Effects Division, Health Canada, Ottawa, Ontario, Canada; 2 Research Division, Canada Mortgage and Housing Corporation, Ottawa, Ontario, Canada

**Keywords:** bacteria, children, endotoxin, housing, respiratory illness

## Abstract

To determine the influence of endotoxin on the incidence of acute respiratory illness during the first 2 years of life, we carried out a longitudinal follow-up study, beginning at birth, of 332 children born in Prince Edward Island, Canada. We measured 5-day averaged air endotoxin in the homes of children, whose parents provided information by daily symptom diaries and twice-monthly telephone contact for up to 2 years. Endotoxin concentration was 0.49 ± 3.49 EU/m^3^ (geometric mean ± geometric SD), and number of annualized illness episodes was 6.83 ± 2.80 (mean ± SD). A doubling of the air endotoxin concentration was associated with an increase of 0.32 illness episodes per year (*p* = 0.0003), adjusted for age, year of study, breast-feeding, environmental tobacco smoke, child care attendance, indoor temperature, and income. Indoor mold surface area and fungal ergosterol were not significantly associated with endotoxin. Airborne endotoxin appears to be a risk factor for clinically symptomatic respiratory illnesses during the first 2 years of life independent of indoor fungus.

Endotoxins are lipopolysaccharide components of the outer membranes of gram-negative bacteria. Endotoxin has been implicated in bysinnosis, organic dust toxic syndrome, and illness in swine confined animal feeding operations workers (Douwes et al. 2002). Endotoxin in settled dust in residential environments has been associated with an increase in asthma symptoms, asthma medications, and reductions in lung function in those with atopy or asthma (Douwes et al. 2000; Gehring et al. 2001a; Michel et al. 1991, 1996; Park et al. 2001a; Rizzo et al. 1997). Despite these adverse effects, early exposure may reduce future allergies and asthma (Lapa e Silva et al. 2000; Litonjua et al. 2002; Reed and Milton 2001; Von Ehrenstion et al. 2000; Von Mutius et al. 2000). Most studies were of adults or school-age children, with two focusing on infants. In the present study we examined the association between airborne endotoxin and the incidence of respiratory illnesses in children during the first 2 years of life. We accounted for exposure to a potential confounder, indoor fungus, which has been associated with respiratory symptoms and may be associated with the presence of indoor endotoxin (Gehring et al. 2001b; Verhoeff and Burge 2004).

## Materials and Methods

### Study design.

Data for the present study were abstracted from an ongoing study of the influence of indoor environmental factors on respiratory illness during the first 2 years of life. The study began in 1997 in the province of Prince Edward Island, Canada, which has a population of approximately 150,000. The study was approved by the ethics review boards of the Ottawa Hospital and the Health Protection Branch of the Canadian government. Recruitment occurred during the late autumn and winter (cold season) of each year when the ground was frozen. Because of resource constraints, we recruited approximately 60 consecutive newborns each year. All physicians who practice obstetrics in the province participated in recruitment. Women in the third trimester of pregnancy received letters from the physicians’ offices describing the study and requesting participation. Interested women were contacted by telephone to obtain informed consent. Excluded from the study were babies born > 4 weeks premature, those with neonatal respiratory difficulties requiring prolonged hospitalization at birth, and those whose families expected to change residence within 2 years of birth. Only one child per household was studied. Baseline information was obtained on sociodemographics and family history. The participating parents maintained a daily symptom diary from birth until 2 years or until the study ended, on large multipurpose calendars. Each study family was phoned twice monthly to document information from the diary. If parents had omitted recording symptoms on a daily basis, they provided information for the previous 2 weeks based on recall. Parental reporting of child care attendance was also recorded every 2 weeks.

### Definition of respiratory illness.

We adapted the method of Samet et al. (1992) to define a respiratory illness episode, the purpose being to identify discrete acute illnesses as opposed to persistent ongoing symptoms, such as a chronically runny nose. We defined the beginning of an illness episode as 2 consecutive days with any one of the four following symptoms: stuffy nose, cough, wheeze, and shortness of breath. The illness episode starts on the first of these 2 consecutive days and ends when there are 2 consecutive days with none of these symptoms, the last day of the illness episode being the last day with a symptom.

The primary outcome of interest was the number of illness episodes prorated on an annual basis (number multiplied by 365/days of observation). A secondary outcome measure, illness days, was defined as the sum of all days occurring within illness episodes, also prorated on an annual basis. For example, if a child had two illness episodes each lasting 3 days, six illness days would be assigned. If a child had two illness episodes each lasting 5 days, 10 illness days would be assigned.

### Air sampling for endotoxin and ergosterol.

Sampling was done within the first year of birth, and for 81%, within the first 4 months. Air from the child’s bedroom was sampled for both endotoxin and ergosterol through a three-piece cartridge equipped with a polycarbonate filter for approximately 5–7 days with a Buck model SS sampling pump (AP Buck, Orlando, FL, USA) calibrated at 2 L/min at the beginning and end of the sampling. Forty-eight hours after the pump was installed, the flow rate was checked to ensure it was within 5% of the initial reading. Very high dust concentrations can clog the filter and reduce the pump flow. If this happened, the pump was stopped and air endotoxin concentration was calculated based on the reduced sampling time. The total volume of air sampled ranged from 6.0 to 23.9 m^3^, limited by the need to maintain an acceptable flow rate. Once collected, the cartridges and filters were sealed in new plastic bags and stored at room temperature under dry conditions.

For endotoxin analysis, the filters were extracted with depyrogenated water (LRW, Associates of Cape Cod Ltd., East Falmouth, MA, USA) assisted by sonication. Samples taken before April 2001 were analyzed by the *Limulus* amoebocyte lysate (LAL) assay gel clot method from Associates of Cape Cod. The detection limit was 0.25 EU/filter. All subsequent analyses were done by the LAL chromogenic method, also from Associates of Cape Cod. The detection limit was 0.1 EU/filter. All analyses were performed by the same analyst in the same laboratory according to the manufacturer’s instructions. Apart from differences in the lower limit of detection, the two methods gave similar results.

We analyzed ergosterol, an indicator of fungal biomass, by gas chromatography and mass spectroscopy as described by Foto et al. (2005). The volume of air sampled ranged from 10.7 to 25.4 m^3^. Ergosterol was determined using an Agilent model 5973 quadrupole mass spectrometer (Agilent Technologies, Inc., Palo Alto, CA, USA) operating in the electron ionization mode at 70 eV. Compounds were separated on an Agilent 6890 series gas chromatograph equipped with a 30 m × 0.25 mm inner diameter × 0.25 μm ZB-5 capillary column. The concentration of ergosterol was determined against an authentic external standard in the selective ion mode using *m*/*z* 363 and 396. The detection limit in selective ion mode was 4.5 ± 0.6 ng/mL. Ergosterol standard (Sigma Chemical Company, St. Louis, MO, USA) was recrystalized, freeze-dried, and stored at 4°C.

### Definition of covariates other than ergosterol.

During the postnatal interviews, several characteristics were recorded every 2 weeks for a period of 2 years: the presence of furry or feathered pets in the house, the presence of smokers inside the house, whether the baby was breast-fed, and the number of hours per week the child was cared for outside the home. Based on this information, we created a summary variable for each of the characteristics. For the first three characteristics, we calculated the percentage of postnatal interviews where the characteristic was declared. For example, if 20 of 50 postnatal interviews mentioned the presence of pets inside the house for a particular child, the value of the pet variable would be 0.4. The last characteristic, the number of hours per week that the baby was cared for outside the home, was averaged over the entire 2-year period to create the child-care variable. For presentation, we then categorized some summary variables. The pet variable was categorized into “never declared pets,” “sometimes declared pets,” and “always declared pets,” which divided responses almost equally into thirds. The exposure-to-smoke variable was categorized into terciles. The breast-feeding and the child-care variables were kept as continuous data. The age variable was defined as the age of the child in the middle of the span of the follow-up period. “Mold surface area” refers to the total surface area of the home with mold visible to trained home inspectors.

### Statistical analysis.

We tested the association between the number of illness episodes per year and airborne bedroom endotoxin concentration using multiple linear regression analysis. Valid endotoxin results were obtained for 351 houses. A total of 19 homes were excluded from the analysis—15 because of missing temperature data and four because of missing income data—leaving 332 homes for analysis. Eleven babies of 332 (3.3%) exited the study before turning 2 years of age (mean age, 1.1 years), and 56 babies had not reached 2 years of age (mean 1.7 years) by the last day of data collection used for this analysis. This is an ongoing study with children entering and exiting at different times. The illness episodes and covariates were annualized and thus adjusted for duration of follow-up. To test the effect of the 11 babies who exited early, we repeated the analysis with and without them, and no differences were found in the illness–endotoxin association.

Mold surface area was expressed as ranks from highest to lowest. Endotoxin values followed a log-normal distribution, so they were log-transformed. A multiple linear regression model with the number of respiratory illness episodes per year as the dependent variable and the natural logarithm of endotoxin concentration as the primary independent variable of interest was built with the stepwise method. A categorical variable—the year of sample collection—was added to the model to account for any seasonal variations in illness from year to year and also the change in the lower limit of detection of the endotoxin analytic technique after 2001. Endotoxin and year of home sampling were held in the model along with any variables with *p*-values < 0.10, resulting in the final model, with variables endotoxin, year of home sampling, temperature, age, mean hour per week that the baby was cared for more than 1 day a week outside the home, percentage of postnatal interviews in which the baby was breast-fed, income, and categorized percentage of postnatal interviews where smokers were declared in home.

A potential confounder was defined as a variable (Tables 1 and 2) that, if added to the model, would change the parameter (β) of the natural logarithm of endotoxin by > 10%. No confounders were found for the illness episodes model. The residuals from the regression equation were normally distributed (Shapiro-Wilk statistic = 0.9932, *p* = 0.1362). We also examined the homogeneity of variance assumption, and the chart of residuals against predicted values showed no particular pattern. Interactions biologically plausible were also tested, and none were found to be statistically significant at the 5% level. We found no evidence of interaction between allergies or asthma in parents and endotoxin. Careful examination of each of the partial residual plots (i.e., the component-plus-residual plot) did not reveal any sign of nonlinearity in the relationship between illnesses and air endotoxin. Further, adding a square term for endotoxin did not improve significantly the *R*^2^ of the model.

The endotoxin measurement was made only at the beginning of the 2-year follow-up. To determine the robustness of the endotoxin–illness association, we measured it at several time points between the initial endotoxin measurement and symptom assessment. We would assume that a true causal association would remain stable or weaken over time. If the association increased or fluctuated randomly with time of follow-up, this would reduce the probability of a causal association. We measured the results from 90-, 180-, 270-, 360-, 450-, 540-, 630-, and 720-day windows around the time of endotoxin sampling. The regression model obtained previously for a 2-year period was applied to each of these windows. The β-coefficient for the effect of the natural logarithm of endotoxin on illness episodes and total illness days along with its 95% confidence interval were graphed against the size of the window.

## Results

The characteristics of the 332 children, overall and stratified by bedroom airborne endotoxin level, are presented in Tables 1 and 2. Of the categorical variables (Table 1), only year of testing was significantly associated with endotoxin, with no secular trends (*p* < 0.0001). The pets variable was not associated with endotoxin concentrations. For dogs, the geometric mean and geometric standard deviation (GSD) were 0.46 ± 3.82 if dogs were never reported present in the home and 0.54 ± 3.15 if ever reported to be in the home (*p* = 0.22). There was also no significant difference in endotoxin values between homes where dogs were reported in < 50% of interviews compared with at least 50% of interviews (*p* = 0.12). Similarly, there was no significant association between cats and endotoxin. Of the continuous variables (Table 2), only indoor relative humidity was positively related to endotoxin (*p* = 0.01).

Illness episodes correlated best with the individual symptoms of cough and stuffy nose; Pearson correlation coefficients were 0.69 and 0.76, respectively, both at *p* < 0.01. For illness days, respective values were 0.83 and 0.95. Wheeze was also significant for both illness episodes and illness days at 0.37 and 0.41, respectively (*p* < 0.01). The annualized number of respiratory illness episodes and total days of illness episodes were positively related to endotoxin at *p* = 0.13 and *p* = 0.07, respectively (Table 3). All of the individual respiratory symptoms were greater in the higher compared with the lower endotoxin group, but only the incidence of wheeze reached statistical significance, being a relative 248% greater in the higher compared with the lower endotoxin group (*p* = 0.01).

The unadjusted Pearson correlation coefficients between log-transformed endotoxin and illness episodes and illness days were 0.105 (*p* = 0.056) and 0.106 (*p* = 0.053), respectively. The association for number of days with wheeze was 0.271 (*p* < 0.0001), but other individual variables were not significant at *p* = 0.05. The adjusted associations for illness episodes and total illness days were highly significant (Tables 4 and 5). The multiple linear regression model for illness episodes resulted in a β-coefficient of 0.46 (SE 0.13) for the natural logarithm of endotoxin (*p* = 0.0003), which means that each 1.0 unit increase in the natural logarithm of airborne endotoxin concentration was associated with 0.46 more illness episodes per year. An alternative expression of the relation would be that a doubling of air endotoxin concentration was associated with an increase of 0.32 illness episodes per year (*p* = 0.0003), adjusted for age, year of study, breast-feeding, environmental tobacco smoke, child care attendance, indoor temperature, and income. Also, starting from the geometric mean (0.49) and increasing endotoxin by its geometric mean resulted in 4.7% excess illnesses per year. Similarly, doubling air endotoxin was associated with an increase of 3.25 illness days per year (*p* = 0.005), adjusted for age, year of study, breast-feeding, child care attendance, indoor temperature, and sex. Starting from the geometric mean (0.49) and increasing endotoxin by its geometric mean resulted in 5.5% excess illness days per year.

Significant covariates in the regression of illness episodes were year of testing, indoor temperature, age, child care, environmental tobacco smoke, and income (all *p* < 0.05). Similar results were found with illness days with a β-coefficient of 4.68 (SE 1.66, *p* = 0.005).

**Figure 1:**
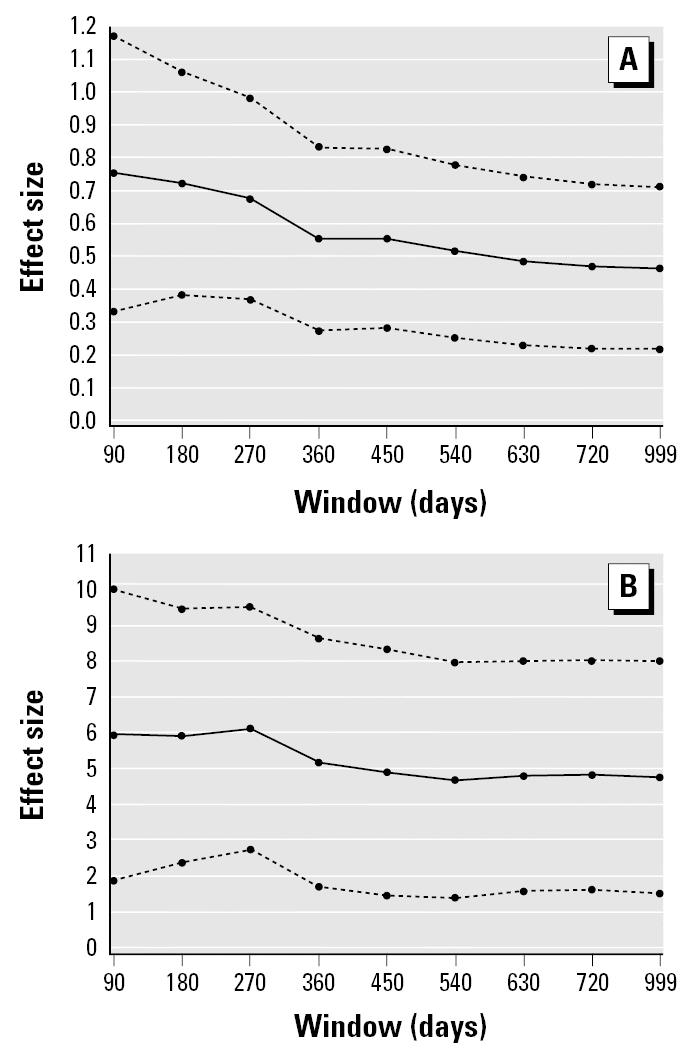
The effect size over time between illness and bedroom endotoxin measured at birth. (*A*) Annualized illness episodes. (*B*) Annualized illness days. Effect size is represented as the β-coefficient for the effect of the natural logarithm of endotoxin on illness along with its 95% confidence interval.

In Figure 1, the β-coefficient for the effect of the natural logarithm of endotoxin on illness episodes and total illness days is graphed against the size of the window. The magnitude of the association between illness episodes and endotoxin levels was almost linearly decreasing with the use of longer observation periods extending further from the original sampling. Because the effect of endotoxin levels on illness episodes was highly significant for a 2-year period, it would be even more significant for shorter observation periods.

## Discussion

Air endotoxin was positively associated with an increase in episodes of respiratory illness among children during their first 2 years of life despite adjustment for many host and environmental factors, including indicators of fungal exposure. The method of endotoxin collection is unique compared with previous studies of indoor air, most of which sampled floor dust rather than airborne dust, which may be better correlated with inhalation exposure. Air sampling, done infrequently in previous studies, usually consisted of a grab sample (up to 30 min), whereas our 5-day collection period would be expected to provide a more stable average estimate of exposure. Another unique feature of exposure is that endotoxin sampling was done during the cold season when the ground is frozen and usually snow covered. This makes it less likely that the measured endotoxin in air is simply a reflection of what was present outdoors at the time of the sampling. Rylander (2002) suggested that fungal products, and specifically (1–3)-β-d-glucan, may coexist with endotoxins and thus may confound the association. In the present study, ergosterol, a marker of fungal growth in Prince Edward Island homes, did not influence the illness–endotoxin association.

Michel et al. (1991) measured endotoxin concentrations in the house dust of 28 adult subjects with chronic stable asthma. Exposure to higher levels (> 5.6 ng lipopolysaccharide/mL) was associated with poorer asthma control measured by symptoms, medication use, and lung function. In a subsequent study, Michel et al. (1996) refined their previous findings in a group of 69 adults with asthma who were sensitized to house dust mites. Asthma control was related to house dust endotoxin, averaging 2 ng/mg dust in those exposed to Der p 1 levels > 10 μg/g dust, but not in those exposed to lower levels of the major dust mite antigen. Rizzo et al. (1997) and Douwes et al. (2000) reported adverse effects of settled dust endotoxin in school-age children with asthma, atopy, or asthma symptoms but not in those without an atopic history. Park et al. (2001a) and Gehring et al. (2001a) reported increased wheezing in infants living in homes with increased levels of settled dust endotoxin, consistent with the findings of the present study in which airborne endotoxin was measured and fungal burden was accounted for using airborne ergosterol. Indoor fungus therefore is not likely to have confounded the observed relation, although fungus has been associated with respiratory symptoms and may be associated with indoor endotoxin (Gehring et al. 2001b; Verhoeff and Burge 2004). Compared with occupational settings, the indoor air endotoxin concentrations in our study were low (on average, < 2 EU/m^3^), yet associations with adverse health effects were observed. The relatively large sample size with daily symptom monitoring over 2 years in each subject appeared to give us the power to detect these effects. Further support from health effects at low levels comes from Park et al. (2000), who reported airborne endotoxin in Boston homes during the warm season to be generally < 1 EU/m^3^, and corresponding floor dust was < 100 EU/mg (estimated values taken from a log–scaled graph). Before our study, Park et al. (2001a) also found associations between settled dust endotoxin and wheeze.

Endotoxins are also postulated to confer health benefits. Lipopolysaccharide, the main component of endotoxin, may shift the cytokine response toward a T_h_1 response and away from a T_h_2 response, thereby reducing the chance of developing atopy (Lapa e Silva et al. 2000). Consistent with this theory is the observation that children growing up on farms, where endotoxin exposure is higher than in urban areas, have less atopy (Von Ehrenstion et al. 2000; Von Mutius et al. 2000). Evidence thus far suggests that early childhood exposure to endotoxin may protect against future asthma, but later in life endotoxin appears to exacerbate asthma (Reed and Milton 2001). The present study indicates that very early exposure is not benign but associated with increased illness episodes. Litonjua et al. (2002) reported on 226 children between the ages of 1 and 5 years with a parental history of atopy who were followed for 4 years. House dust endotoxin was associated with reported wheezing that decreased with increasing duration of follow-up. This observation suggests that early exposure offers future protection, or that the initial endotoxin measure became less representative of ongoing endotoxin exposure over time. The present study was somewhat different: beginning at birth, including all children irrespective of parental history of atopy, using airborne rather than dust endotoxin, including indicators of respiratory illness in addition to wheeze, and considering confounding by indoor mold exposure. Nevertheless, even with children not selected based on atopic parents, we also found that wheeze was the symptom with the strongest association with endotoxin, and the effect size became smaller with increased duration of follow-up.

### Sources of endotoxins.

Gram-negative bacteria are found in water, soil, and outdoor air. Reported indoor sources of gram-negative include contaminated humidifiers, pets, storage of food waste, and increased amounts of settled dust (Park et al. 2001b). The need for water availability is consistent with our finding that relative humidity was positively associated with air endotoxin, not previously described. Gehring et al. (2001a) found that dust concentrations were higher with cats and dogs present. These studies found that endotoxin was higher in old buildings, with longer duration of occupancy, low ventilation rate, and poor housekeeping. Indoor pets were not associated with air endotoxin in the present study, which was carried out during the cold season with frozen ground and often snow cover. Perhaps pets would be less likely to go outside and subsequently bring in soil on their paws.

In summary, the present study supports a positive association between airborne endotoxins and the incidence of acute respiratory illnesses during the first 2 years of life, independent of allergic history and exposure to indoor mold that may coexist with contamination by bacterial endotoxin.

## Figures and Tables

**Table 1:** Characteristics of 332 children overall and stratified by terciles of endotoxin for categorical variables [no. (%)].

Characteristic	Overall	1st tercile	2nd tercile	3rd tercile	*p*-Value^a^
Male sex	167 (50.3)	53 (47.8)	54 (49.1)	60 (54.1)	0.61
Parent with asthma or allergies^b^	174 (52.7)	64 (57.7)	50 (45.9)	60 (54.6)	0.19
Parent with university education	197 (59.3)	66 (59.5)	64 (58.2)	67 (60.4)	0.95
Family income					0.61
< $30,000	67 (20.2)	19 (17.1)	20 (18.2)	28 (25.2)	
$30,000–49,999	114 (34.3)	39 (35.1)	39 (35.5)	36 (32.4)	
≥$50,000	151 (45.5)	53 (47.8)	51 (46.4)	47 (42.3)	
Environment					
Furry or feathered pets					0.70
Never	86 (25.9)	33 (29.7)	24 (21.8)	29 (26.1)	
Sometimes	126 (38.0)	42 (37.8)	42 (38.2)	42 (37.8)	
Always	120 (36.1)	36 (32.4)	44 (40.0)	40 (36.0)	
Exposure to smoke					0.73
Low	114 (34.3)	33 (29.7)	40 (36.4)	41 (36.9)	
Medium	110 (33.1)	40 (36.0)	33 (30.0)	37 (33.3)	
High	108 (32.5)	38 (34.2)	37 (33.6)	33 (29.7)	
Year tested					< 0.0001
1998	52 (15.7)	9 (8.1)	15 (13.6)	28 (25.2)	
1999	53 (16.0)	8 (7.2)	19 (17.3)	26 (23.4)	
2000	45 (13.6)	21 (18.9)	14 (12.7)	10 (9.0)	
2001	58 (17.5)	36 (32.4)	13 (11.8)	9 (8.1)	
2002	58 (17.5)	24 (21.6)	14 (12.7)	20 (18.0)	
2003	66 (19.9)	13 (11.7)	35 (31.8)	18 (16.2)	

^a^*p*-Value of the Pearson chi-square test of association between the characteristic and endotoxin.

^b^There are two missing values for parent with asthma or allergies.

**Table 2:** Characteristics of children’s home environments overall and stratified by terciles of endotoxin.

	Overall		1st tercile		2nd tercile		3rd tercile		
Variable	No.	Mean ± SD	No.	Mean ± SD	No.	Mean ± SD	No.	Mean ± SD	*p*-Value^a^
Children									
Age (days)	332	351 ± 42.1	111	355 ± 34.9	110	337 ± 59.0	111	360 ± 19.6	0.38
Mean hours/week child care	332	7.89 ± 8.97	111	8.43 ± 9.18	110	7.65 ± 8.48	111	7.60 ± 9.28	0.49
Percent postnatal interviews where breast-feeding reported	332	30.1 ± 26.7	111	31.0 ± 25.4	110	29.2 ± 29.5	111	29.9 ± 25.1	0.78
Environment									
Endotoxin (EU/m^3^)	332	0.49^b^ ± 3.49^c^	111	0.14^b^ ± 2.32^c^	110	0.50^b^ ± 1.32^c^	111	1.80^b^ ± 2.10^c^	—
Living room ergosterol (ng/m^3^)	319	0.15^b^ ± 4.12^c^	107	0.16^b^ ± 3.94^c^	105	0.16^b^ ± 3.84^c^	107	0.14^b^ ± 4.63^c^	0.40^d^
Bedroom ergosterol (ng/m^3^)	319	0.14^b^ ± 4.24^c^	106	0.14^b^ ± 4.14^c^	105	0.15^b^ ± 3.93^c^	108	0.14^b^ ± 4.71^c^	0.81^d^
Temperature (°C)	332	20.9 ± 2.32	111	20.9 ± 2.24	110	20.7 ± 2.15	111	21.0 ± 2.33	0.97
Relative humidity (%)	332	31.5 ± 6.24	111	30.8 ± 5.74	110	31.5 ± 5.80	111	33.0 ± 6.52	0.01
Interior wood storage (m^3^)	332	1.54 ± 4.39	111	1.12 ± 3.82	110	1.81 ± 5.01	111	1.70 ± 4.26	0.33
Mold area rank (%)	332	51.3 ± 29.3	111	50.3 ± 29.1	110	51.4 ± 30.5	111	50.7 ± 28.2	0.93

^a^*p*-Value of the Fisher test for a linear trend for the terciles of endotoxin.

^b^Geometric mean.

^c^Geometric SD.

^d^*p*-Value of the Fisher test for a linear trend for the terciles of endotoxin using the natural logarithm of the variable.

**Table 3:** Incidence of children’s illness overall and stratified by terciles of endotoxin concentrations.

	Overall		1st tercile		2nd tercile		3rd tercile		
Variable	No.	Mean ± SD	No.	Mean ± SD	No.	Mean ± SD	No.	Mean ± SD	*p*-Value^a^
No. of episodes of any illness^b^ per year	332	6.83 ± 2.80	111	6.66 ± 2.86	110	6.60 ± 2.65	111	7.22 ± 2.85	0.13
No. of illness days per year	332	58.5 ± 36.4	111	54.6 ± 34.4	110	57.5 ± 36.4	111	63.5 ± 38.0	0.07
No. of days with cough per year	332	33.0 ± 25.3	111	31.9 ± 23.8	110	31.0 ± 24.4	111	36.1 ± 27.6	0.21
No. of days with wheeze per year	332	4.06 ± 9.66	111	2.28 ± 4.55	110	4.26 ± 10.1	111	5.66 ± 12.4	0.01
No. of days with SOB per year	332	0.41 ± 1.20	111	0.30 ± 0.99	110	0.48 ± 1.25	111	0.46 ± 1.33	0.30
No. of days with stuffy nose per year	332	48.7 ± 31.2	111	46.1 ± 29.7	110	48.1 ± 31.3	111	51.9 ± 32.7	0.17

SOB, shortness of breath.

^a^*p*-Value of the Fisher test for a linear trend for the terciles of endotoxin.

^b^Illnesses are cough, wheeze, shortness of breath, and stuffy nose. See “Materials and Methods” for details on how we defined illness episodes.

**Table 4:** Association between illness episodes (dependent variable) and natural logarithm of endotoxin concentrations: multiple linear regression analysis.

Independent variable	β	SE	*p*-Value
Unadjusted (model *R*^2^ = 0.01)			
Intercept	6.99	0.18	< 0.0001
Ln(endotoxin)	0.24	0.12	0.0556
Adjusted (model *R*^2^ = 0.18)			
Intercept	7.74	1.92	< 0.0001
Ln(endotoxin)	0.46	0.13	0.0003
Year tested			0.0001
1998	−0.77	0.55	0.1645
1999	−1.23	0.55	0.0269
2000	−0.37	0.56	0.5019
2001	1.18	0.55	0.0321
2002	−0.90	0.52	0.0881
2003	Reference		
Temperature	−0.21	0.06	0.0012
Age	0.01	0.004	0.0038
Breast-feeding	0.94	0.58	0.1031
Child care	0.04	0.02	0.0200
Exposure to smoke			0.0483
Low	−0.70	0.39	0.0757
Medium	0.17	0.37	0.6358
High	Reference		
Income			0.0204
< $30,000	−0.57	0.41	0.1614
$30,000–49,999	−0.93	0.33	0.0056
≥$50,000	Reference		

**Table 5:** Association between illness days (dependent variable) and natural logarithm of endotoxin concentrations: multiple linear regression analysis.

Independent variable	β	SE	*p*-Value
Unadjusted (model *R*^2^ = 0.01)			
Intercept	60.7	2.28	< 0.0001
Ln(endotoxin)	3.09	1.59	0.0533
Adjusted (model *R*^2^ = 0.15)			
Intercept	47.3	24.7	0.0565
Ln(endotoxin)	4.68	1.66	0.0050
Year tested
1998	−1.50	6.90	0.8279
1999	−4.88	7.01	0.4863
2000	4.77	7.08	0.5003
2001	13.2	7.17	0.0674
2002	−7.77	6.78	0.2528
2003	Reference		
Temperature	−2.73	0.85	0.0014
Age	0.16	0.05	0.0029
Breast-feeding	0.70	0.22	0.0013
Child care	18.3	7.45	0.0147
Sex (male)	7.36	3.79	0.0533
